# Passive and Self-Powered Autonomous Sensors for Remote Measurements

**DOI:** 10.3390/s90200943

**Published:** 2009-02-13

**Authors:** Emilio Sardini, Mauro Serpelloni

**Affiliations:** Department of Electronics for Automation University of Brescia / V. Branze 38, 25123 Brescia, Italy; E-Mail: emilio.sardini@ing.unibs.it

**Keywords:** Autonomous sensors, power harvesting, energy scavenging, contactless sensors, telemetry system, self-powered sensors, wireless sensors

## Abstract

Autonomous sensors play a very important role in the environmental, structural, and medical fields. The use of this kind of systems can be expanded for several applications, for example in implantable devices inside the human body where it is impossible to use wires. Furthermore, they enable measurements in harsh or hermetic environments, such as under extreme heat, cold, humidity or corrosive conditions. The use of batteries as a power supply for these devices represents one solution, but the size, and sometimes the cost and unwanted maintenance burdens of replacement are important drawbacks. In this paper passive and self-powered autonomous sensors for harsh or hermetical environments without batteries are discussed. Their general architectures are presented. Sensing strategies, communication techniques and power management are analyzed. Then, general building blocks of an autonomous sensor are presented and the design guidelines that such a system must follow are given. Furthermore, this paper reports different proposed applications of autonomous sensors applied in harsh or hermetic environments: two examples of passive autonomous sensors that use telemetric communication are proposed, the first one for humidity measurements and the second for high temperatures. Other examples of self-powered autonomous sensors that use a power harvesting system from electromagnetic fields are proposed for temperature measurements and for airflow speeds.

## 1. Introduction

Autonomous sensors can be defined as devices that autonomously execute their measurement functions in the measurement environment. They are also unwired from the acquisition unit; they are characterized by autonomous power supplies and the ability to measure and transmit data. They can achieve different functionalities ranging from simple detectors, giving an alarm signal when the sensor passes a threshold, up to monitoring systems collecting measurement data of different physical or chemical quantities. Autonomous sensors are increasingly used in many applications, mostly in measuring physical phenomena. They can be applied for measurement of quantities both in mobile devices, or in protected environments, or in spaces where electrical energy is absent. Their use widens also to applications where wires connecting a data acquisition unit and the sensor element cannot be used such as, for examples, in implantable devices inside the human body to avoid risk of infections or skin damage [1-3] in rotating machinery, [4], or in hermetic environments [5]. In the industrial field a cable connection of the machine produces friction, stiffness and damping, limiting movement. The cables can be easily damaged, which affects the reliability of the measurement system. Hermitically sealed bags are essential for dry foods such as potato chips and various types of cereals to retain their freshness and safety. Autonomous sensors can improve the current shelf life labels by letting both consumers and producers know when the packaged food is fresh and safe. In the food logistics field autonomous sensors are related to the product and follow it along all the food chain, acquiring data and registering the crossing of several thresholds in terms of temperature, humidity, light or gas concentrations [6-7]. In the biomedical field cable connections limit the patient's mobility and, moreover, may cause skin irritations or infections. Some applications of autonomous sensors can be founded in remote monitoring apparatus for the measurement and recording of physiological parameters [8-11]. Autonomous sensors are applied on live animals for analysis of brain stimulants to analyze neurochemical data for research purposes. These systems are small and light enough to record biopotentials from awake birds and insects. This technique allows, for example, real-time reading of glucose levels in diabetic patients, critical care and brain injuries. In orthopedic science autonomous sensors are used for accurate measurements of knee forces in total knee arthroplasty [1]. These forces produce wear in polyethylene, stress distribution in the implant and the implant–bone interface, and stress transfer to the underlying bone.

Autonomous sensors are adopted in many other fields: in the literature applications in harsh environments are described, such as under high temperatures, cold, humidity or corrosive conditions [12-17]; applications in which long distances are to be bridged or a big number of distributed components is necessary, such as smart homes, environmental applications [18] or mobile applications for the monitoring of environmental conditions [19]. Common examples of applications are the structural health monitoring of bridges or buildings [20] and the monitoring of climate conditions or pollution [21]. In environmental monitoring flow and temperature are important parameters for efficient control of domestic or industrial plants [22]. In these cases, temperature values along the sections of a heating or cooling plant are important indicators to control the energy efficiency in the regulation of thermal comfort [23-24].

Usually an autonomous sensor requires a power source: several examples reported in literature are equipped with batteries, but other power sources are emerging such as: harvesting modules and inductive links. Since the voltage and current levels of the electronic circuits do not currently meet the possibility offered by power harvesting system or sometimes even by batteries, management of the power supply is required; this block commonly consists of a dedicated DC-DC converter and power supervision circuits. Several sensors are powered by rechargeable batteries [18-19]. However, batteries frequently dominate the size and weight of the device. Batteries introduce unwanted maintenance burdens of replacement and, they often cannot be easily replaced since the autonomous sensor is placed in a protected environment. Moreover, the disposal of the increasing number of batteries is creating an important environmental impact as they contain toxic chemicals.

Since autonomous sensors are wireless devices, they encounter the typical problems of a wireless network. If the distance between the wireless device and the data collection system is short, a point to point communication can be implemented. Point to point communication avoids the integration into the autonomous system of circuits to manage the complexity of a network protocol, saving power and making the system compatible with the available low energy. Point to point communication exploits an ID code that can be assigned to every autonomous sensor with the aim of univocally individuating the device. This principle is implemented in RFID technology in particular. Nowadays several RFID communication standards exist, with different working ranges and data rates, which are applied to different applications.

In this paper some autonomous sensors working without batteries are presented and discussed. A classification of autonomous sensors into “passive autonomous sensors” and “self powered autonomous sensors” is introduced. “Passive autonomous sensors” are defined those that are just passive elements, interrogated wirelessly by a readout unit. “Self-powered autonomous sensors” are those that have a power-harvesting module or are supplied power by an electromagnetic field. In the next section the general architectures of passive and self-powered autonomous sensors are described and discussed.

## 2. Architectures of Autonomous Sensors

**Figure 1.**
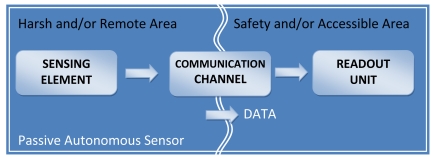
Block diagram of a passive autonomous sensor.

A general architecture of a measurement system based on a passive autonomous sensor is shown in Figure 1. The passive autonomous sensor is the sensing element in the harsh or remote area, while the readout unit is placed in the safety zone. The two elements are connected by a wireless communication exploiting an electric-magnetic, optic or acoustic link. Between the sensing element and the readout unit there is usually a barrier whose characteristics (mainly material and geometry) influence the system's performance. The sensing element is a passive device that does not require any power supply. The quantity under measurement is usually seen as reflected impedance by the front-end electronics contained into the readout unit.

Some sensing devices can be classified as passive autonomous sensors: examples are quoted in [13, 25-32]. In [25] a NiFe sensor is associated to a remote magnetic transducer and provides a contactless temperature measurement with a readout distance of about 4 mm. In [26], LED based chemical sensors use passive elements constituted by chemical sensing materials placed in the harsh environment. These elements are remotely interrogated through transmittance and reflectance absorptiometric measurements. In [27] a magnetostrictive cantilever coupled with a bio-recognition element is remotely actuated and sensed using magnetic signals. Most passive autonomous sensors use a telemetric communication constituted by two inductors, one connected to the sensitive element (in the following referred as “readout inductor”), and the other to the measuring circuit [13, 28, 30]. In [28] a system for environmental wireless monitoring consists of a LC sensor and two loop antennas (transmitter and receiver). A change of the L and/or C parameters is reflected as mutual impedance on the receiver antenna. In the one antenna monitoring approach the distance from sensor to readout unit (15 cm) is influenced by the antenna size (single-turn loop with a radius of 4 cm) and the transmitting power level (10 dBm). In [30] the coil core of a wire wound inductor is a micromachined capacitive pressure device; the sensor operates in harsh or protected environments and can be remotely interrogated by a wireless set-up. The autonomous sensor has been tested in a plastic chamber full of water; the resonant frequency of the tank is monitored outside by an antenna connected to an impedance analyzer.

In the literature different techniques to measure the resonance of a telemetric system are used. The first method measures the frequency at which the phase of the impedance reaches its minimum (Min-phase method) [29]. The second method measures the frequency at the maximum of the real impedance (resistance), and the frequency where the imaginary impedance (reactance) is at zero [13]. In the recent years a more accurate method measures three resonances, compensating the distance variation between the two inductors (3-Resonancies Method) [31-32].

**Figure 2.**
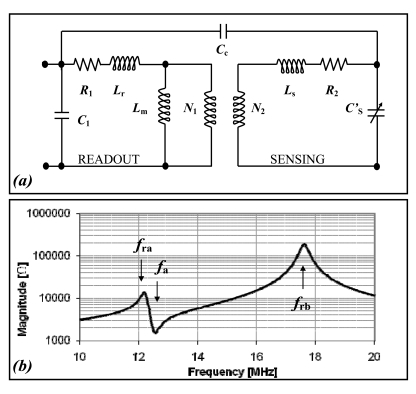
**(a)** Physical model of an autonomous sensor. **(b)** Module and phase of the impedance as seen from the terminal of the readout [32].

A model of an inductive telemetric system is illustrated in Figure 2(a). The parameters have the following meaning: R_1_, R_2_ are the equivalent resistances of readout and sensor; C_1_, C′_S_ are the parasitic capacitances of the readout and sensor; L_r_, L_s_ are the readout and sensor leakage inductances; L_m_ is referred to coupled flux; N_1_ and N_2_ are the equivalent number of the inductor windings; C_c_ is the coupling capacitance. The impedance as seen from the terminal of the readout inductance is qualitatively plotted in Figure 2(b), which shows the three resonant frequencies (*f*_ra_, *f*_rb_, *f*_a_). According to the 3-Resonancies method, the sensor and the parasitic capacitance (C′_S_) can be calculated by:

(1) $$C_{\text{S}}^{\prime }=\frac{L_{1}C_{1}}{L_{2}}\frac{{\left(2\pi f_{\text{ra}}\right)}^{2}+{\left(2\pi f_{\text{rb}}\right)}^{2}-{\left(2\pi f_{\text{a}}\right)}^{2}}{{\left(2\pi f_{\text{a}}\right)}^{2}}$$

The value of C′_S_ is obtained as the product among a constant term and one calculated by the measures of *f*_ra_, *f*_rb_, *f*_a_. The constant term can automatically be obtained by calibration, or calculated by measuring the parameters of the equivalent circuit of every single inductor: L_1_ and L_2_ represent the inductance values of the readout and sensing inductors.

**Figure 3.**
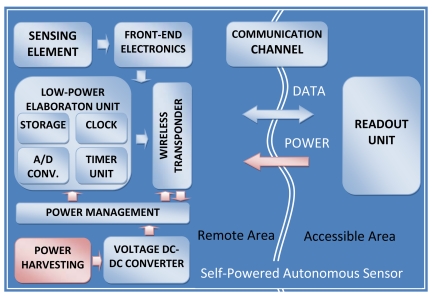
Block diagram of self-powered autonomous sensors.

Differently from passive autonomous sensors, self-powered autonomous sensors are autonomous devices having the capability and functionality of a stand-alone measurement unit, even if the readout unit is not close. Self-powered autonomous sensors should be able to execute measurements, store the measurement data and send these values to the readout unit. In literature examples of self-power autonomous sensors are being increasingly reported. Many examples [5, 11, 33] use batteries as the power source. In [5], physical and chemical sensors for logistic data-logging applications to evaluate food quality and freshness are discussed. In [11] recent results of autonomous sensor research for brain stimulation and neuronal activity recording are reported. In [33] an autonomous sensor monitored patient vital sign data in a hospital. Other examples of autonomous sensors that do not use batteries are quoted in [23-24, 34-37]; all the internal modules are supplied by a power harvesting module or by the electromagnetic field of a wireless link. Since the possibility of substituting batteries with harvesting system is greatly attractive from an ecological point of view, our analysis will be concentrated only on autonomous sensors equipped with harvesting systems. These self-powered autonomous sensors consist of one or more sensing elements and different modules: front-end electronics, an analog-to-digital converter, an elaboration unit to manage the internal tasks, power management, a wireless transceiver and storage memories. In Figure 3 a block diagram of a self-power autonomous sensor is shown. Common characteristics can be extracted: very low-power design, stand-alone configuration, minimal control and communication circuits in order to achieve the smallest and most easily attachable form.

Self-powered autonomous sensors require specific level of voltage and current supply obtainable by an appropriate power management block. Usually the power management circuit has a dedicated DC–DC converter or charge pump to match the output electric impedance of the generator with the characteristics of the circuit load realizing a maximum power transfer. In [24] a characterization of thermoelectric modules connected to a charge pump has been described. Since the power harvesting can work intermittently or the autonomous sensor can require much higher energy than that available from the power harvesting block, energy storage elements can be useful. In the literature different emerging types of energy harvesting for small-scale devices are reported: thermoelectric, vibration-to-electric, and radiofrequency RF power conversion [23-24, 34-37]. Table 1 shows a comparison of some published power harvesters for self-power autonomous sensors.

Different characteristics are indicated: the calculated values are estimated in accordance with the data reported in the referenced papers. As it can be deduced from the Table, an autonomous sensor can rely on a budget of less than 1 mW from a generic power harvesting system. Furthermore, the power supply can be harvested from the magnetic field (or electromagnetic field) generated by the readout unit [12-17]. In this case the inductive telemetric links are often used for transmitting power, data or both. The remote power is transferred through the unique couple of inductors. In [38] a wireless transponder for data and power transmission is reported, during the transmission the power consumption is about 900 μW. A lower value of 810 μW can be found in [39]. Other examples of typical data and power transmissions are passive RFID devices.

Reducing power consumption is a strategy to match the amount of energy available from the power supply devices. Firstly, sensors implement low-power transducing techniques, mostly capacitive or inductive, or use some specific MEMS structures devoted to reduce the power demand. In [5] the MOX sensor is an array of four micro-hotplates with a 80 μm circular active area and it consumes 8.9 mW at continuous 400 °C operation. Secondly, integrated electronic circuits are very low power: the control unit implements particular control strategies triggering periodic activity and long intervals during which the autonomous sensor sleeps. The consequence is that the active process flow could be: wake up, sensor activation, sample acquisition, measurement, computation, data storage and/or transmission, sensor reset, and sleep. A benefit is obtained if the power required for turning off and on of the sensor is much less than that saved during the powering off of the sensor. Obviously the active time should be as short as possible due to the power consumption and the frequency of the main control cycle should be as low as possible. These requirements can be in conflict with the characteristics of the measurement process where high acquisition frequency is usually a system requirement. In [40] a generic sensor interface chip (GSIC) for capacitive sensors is discussed. The device has an averaged consumption of 48 μW in a monitoring system, which contains a sensor, a microcontroller and a wireless layer. The elaboration unit controls the sensor interface circuit, configures the readout electronics and converts the data coming from the sensor interface circuit and stores it in a memory. In order to reduce the energy consumption for the data transmission, it also can implement some smart compression algorithms on the measurement data. Lastly, it drives the telemetric communication with the readout unit. Furthermore, all the internal modules, which are not in use, can be switched off individually. In [41] the power consumption of the autonomous sensor during a cycle is about 60 μW, ideally synchronized and with a measurement interval of about 8 s. Usually the power required to transmit the data depends also on the distance reached, the data throughput and the frequency transmission. An increase in the values of the above parameters causes a corresponding increase in the power consumption.

## 3. Passive Contactless Autonomous Sensors

Two examples of passive autonomous sensors designed and tested in our laboratories are presented in the following sections. They use telemetric communication and one has distance compensation capability. The distance covered by the telemetric communication is about 30 mm.

### 3.1. Passive Autonomous Sensor for humidity measurements

**Figure 4.**
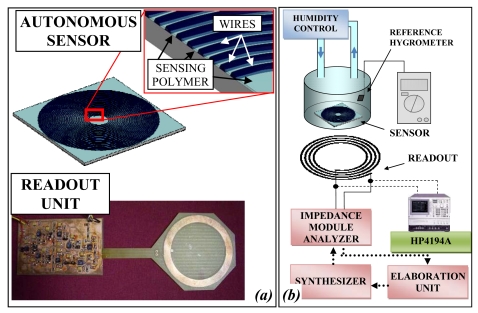
(a) Picture of the passive autonomous sensor and readout system for RH measurement and (b) block diagram of the experimental set-up.

Measurements of relative humidity (RH) in hermetic environments, for example in logistic and biomedical fields, can be executed wirelessly by passive autonomous sensors. In Figure 4(a) an example of autonomous sensor and readout system for this application are schematically represented.

**Figure 5.**
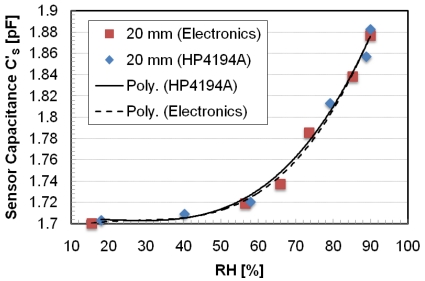
The calculated capacitance values as a function of RH and for different distance values.

**Figure 6.**
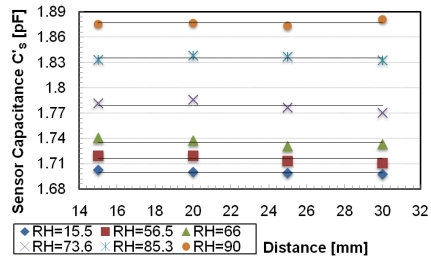
Capacitance values calculated using the proposed conditioning electronics as a function of RH and distance.

The passive autonomous sensor is a standalone planar inductor, fabricated in PCB technology of 25 windings with an external diameter of 50 mm covered by polyethylene glycol (PEG). The relative humidity (RH) variations change the dielectric of the polymer deposited over the inductor causing a variation of the parasitic capacitance. According to the technique developed in [31], a conditioning circuit individuates the three resonant frequencies and a microprocessor calculates the RH and compensates the distance variation. The readout system consists of different functional blocks: one generates the sinusoidal reference signal, the second measures the impedance module and the third calculates RH. The sensor has been characterized using the experimental setup shown in figure 4(b) and discussed in [32]. The sensor is positioned inside a Plexiglas chamber, which is used as a hermetic container for damp air. In the chamber there is a hygrometric sensor (HIH-3610) for reference measurements. The inductances are positioned parallel and their axes are coincident. The three resonant frequencies are monitored by an impedance analyzer (HP4194A), connected to the readout inductor or alternatively, to the dedicated electronics. The damp air that flows inside the chamber is produced by the system that controls the mixture of the two gaseous fluids by two flux-meters. The distance of the readout from the sensor is controlled by a micrometric screw with resolution 10 μm and runs up to 25 mm. The three resonant frequencies have been measured at a distance of 20 mm and, according to the 3-Resonancies Method [31], the calculated capacitance values are reported as square points in Figure 5. In the same figure the capacitance values obtained using an impedance analyzer (HP4194A) instead of the electronics of the autonomous sensor are reported as rhombuses. All the measurement points are a function of the RH values as measured by the reference sensor. Interpolating the two sets of measurement data the maximum difference between the two curves is less than 15 fF, corresponding to less than 8% of the capacitance measurement range. In figure 6 the capacitance values as a function of distance are reported over a distance variation from 15 to 30 mm. The maximum variation of the capacitance is, in the worst case, limited to 20 fF corresponding to about of 1% of FS for each millimeter of distance variation.

### 3.2. Passive Autonomous Sensor for high temperature measurements

**Figure 7.**
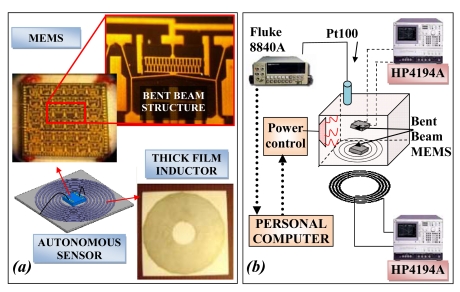
(a) Sketch of the passive autonomous sensor for high temperature measurement and (b) block diagram of the experimental set-up.

A passive autonomous sensor for high temperature measurement is represented schematically in Figure 7(a): the sensor is a hybrid MEMS composed by a novel MEMS temperature sensor (above in Figure 7a) developed using the Metal MUMPs process [42] and a planar inductor (below in Figure 7a) realized in thick film technology by screen printing over an alumina substrate a conductive ink in a spiral shape.

The MEMS temperature sensor exploits a cascade of 36 bent beam structures. The single structure is composed by a V-shaped beam anchored at two ends as reported in the enlargement in the upper part of Figure 7(a). The temperature variation induces a thermal expansion of the structure generating a displacement of the central apex, which is connected to an interdigitated comb. The device is built directly over a silicon nitride isolation layer with a nickel and gold structural layer. The maximum operating temperature is due to the maximum operating limit of nickel (350 °C). The MEMS capacitor is coupled to an embedded coil inductor and the equivalent LC circuit has a resonance frequency which depends on the temperature to be measured. An external inductor can be applied to the external part of the barrier delimiting the harsh zone. The two inductors represent an inductive telemetric system. In the experimental set-up the oven, in which the autonomous sensor is positioned, has a windows of tempered glass with a thickness of 8 mm.

Figure 7(b) shows a block diagram of the experimental setup. In the measurement chamber (in the center of the figure) an IR heater of 500 W rises the temperature up to 350 °C. Three Pt100 thermo-resistances (only one is shown in the Figure) measure the internal temperature in three different points, and each one is connected to a multimeter (Fluke 8840A). The three values are used to assure that the temperature is uniformly distributed. A Personal computer, over which runs a developed LabVIEW™ virtual-instrument, is connected to the multimeters through an IEEE 488 bus and to the input of the power control through the digital output of the I/O board. The PC monitors the temperature inside the oven and controls the IR heater by turning alternatively on and off the power circuit. Two MEMS sensors are placed in the oven. The first one is directly connected to the impedance analyzer (HP4194A) to measure its capacitance, the second one is connected to the planar inductor for the telemetric measurement. Externally the readout inductor is connected to a second impedance analyzer (HP4194A).

**Figure 8.**
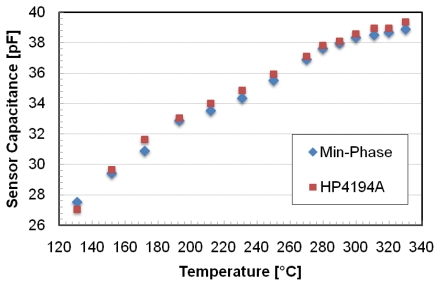
Sensor capacitance directly measured by HP4194A and the data obtained by the telemetric system.

In Figure 8 the sensor's capacitance measured at 2 MHz is reported as a function of the temperature: square points are the values directly measured on the sensor terminals, while the others are measured from the external inductor and calculated using the Min-phase method [29]. The diagram shows a quasi linear behavior of the sensor. The values calculated with the Min-phase method are closely to the reference one measured with the impedance analyzer (HP4194A).

## 4. Self-Powered Autonomous Sensors with Thermoelectric or Airflow Generator

**Figure 9.**
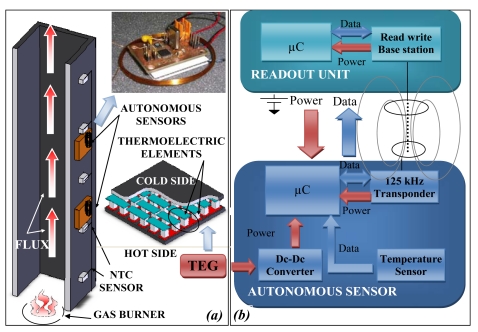
(a) The experimental set-up of the self-powered autonomous sensor for temperature measurement and (b) the block diagram of the autonomous sensor and readout system.

Two self powered autonomous sensors are presented in this paragraph, one measures the temperature and the second the wind velocity. They use a thermoelectric and an electromechanical harvesting system. In the second example the measured parameter is supplied by the same energy used by the power harvester. Temperature values along the section of heating plant are important indicators to control the energy efficiency in the regulation of the thermal comfort [23-24]. An autonomous sensor system consisting of a low power microprocessor, a 125 kHz RF-ID transponder, a low-power temperature sensor and an energy harvesting module has been developed for temperature measurement of walled-in pipes [24]. Figure 9 reports in (a) the experimental set-up for the testing of the self-power autonomous sensors, while in (b) the block diagram of the sensor and readout system.

If the autonomous sensor is placed on the hot pipes, a thermoelectric generator harvests energy, powering the autonomous sensor that periodically performs the temperature measurement and saves the data in non-volatile memory. A time stamp associated to the single data can be saved, but if the thermal gradient is not sufficient to guarantee continuously the power on, it can be lost, but the data are not overwritten. When the remote unit is close to the autonomous sensor, it generates an electromagnetic field exploited by the autonomous sensor to power its circuits and to communicate the stored measurement data to the same remote unit. In this way the autonomous sensor harvests two types of energy always available for the function it has to execute: it measures and stores the temperature when the same temperature is high, and thermal energy is available and communicates the stored data when the remote unit is close.

The thermoelectric generator produces electrical power directly from temperature differences using the Seebeck effect. When a temperature difference ΔT is applied between the TEG faces, an open-circuit output voltage V_G_ is generated according to the following equation:

(2) $$V_{G}=N\alpha \Delta T$$

where α is the Seebeck coefficient of the TEG materials (368 μV/°C), N is the number of thermocouples (254) and ΔT is the temperature difference applied. The used TEG is the module TGM-254-1.0-1.3 by Kryotherm with dimensions of (40×40×3.6) mm^3^.

The output power delivered by the thermoelectric module to the load depends on the difference between the heat rates that flow from the waste heat source to the hot junctions and from the cold junctions to the environment. On matched-load conditions, the output power and the output voltage are about 27 mW and 0.9 V, respectively, with a temperature difference of about 9 °C. The thermoelectric generator output is directly connected to the DC-DC converter (TPS61200) that assures the voltage level required by the electronic circuits. During the measuring and saving data operations the current consumption is about 400 μA at 2.1 V, corresponding to about 840 μW. While, during the telemetric communications, the current consumption of the microprocessor (9S08QE128), the sensor (LM94022) and the transceiver (U3280M) is about 220 μA at 2.58 V, corresponding to about 570 μW.

**Figure 10.**
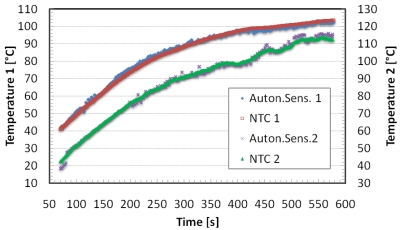
Autonomous sensors measured temperature and reference NTC measured temperature.

An experimental set-up described in [23] has been arranged to test the developed autonomous sensors. The experimental system consists of a flue, in which hot air is fluxed throughout. The heater system represents a simplified model of a generic building heating plant. The hot air is conveyed into a metallic pipe, heating its surfaces. The pipe is made of enameled iron and has a thickness of 1 mm; a square cross section with sides of 100 mm and a length of 1 m. External temperature distribution along the flue was measured using five NTC thermistors placed every 20 cm from the lower end of the pipe. Two autonomous sensors were placed on the external side of the flue respectively 20 and 60 cm from the lower end of the pipe, near NTC sensors. During the test the temperature measured by both the reference and autonomous sensors, the voltage generated by thermoelectric generators have been evaluated. Figure 10 shows the temperature values as a function of time. The experimental results show the functioning of the system during measuring, saving and data transfer operations. The temperature data measured by the autonomous sensors agrees with those of the reference sensors being the maximum temperature difference of about 3.4 °C.

**Figure 11.**
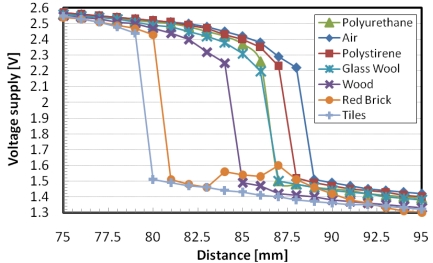
Transponder Voltage Supply for different distances and materials.

The telemetric communication has been characterized interposing between the readout system and the autonomous sensor layers of different material. Metallic layers have been tested as well, but as expected, they do not permit the communication. The tested materials are: polystyrene (thickness 6 cm), polyurethane (6 cm), wood (4 cm), glass wool (4 cm), red brick (5 cm) and tiles (4 cm). In Figure 11 the voltage generated by the transponder is measured as a function of the distance between the autonomous sensor and remote unit. The normal working operations are executed with transponder voltage supply over 1.8 V. As expected, the air curve presents the highest readout distance, while the other curves present a readout distance of few centimeters less.

**Figure 12.**
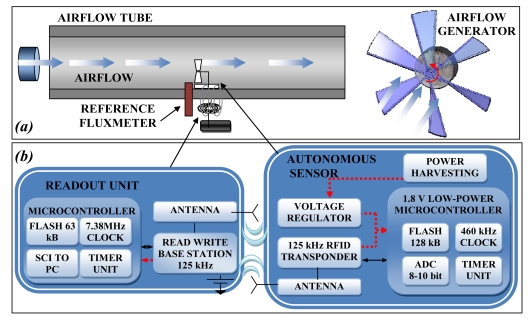
(a) The experimental set-up of the self-powered autonomous sensor for flow measurement and (b) the block diagram of the autonomous sensor and readout unit.

In several environments modest ambient flows are present, for example, in air-conditioning ducts, in outdoor environment, or in moving vehicles. A flow measurement is an important indicator to control the energy efficiency in the regulation of the conditioning implants as well [22]. An autonomous sensor placed inside the pipes and powered by an electromechanical generator scavenging energy from the airflow has been designed and tested (Figure 12a). The adopted block diagram (Figure 12b) is similar to that of the autonomous sensor for temperature monitoring described above, while the airflow is measured trough the rotor frequency of the electromechanical generator. Moreover, when the readout unit is active the electromagnetic field is used to power the autonomous sensor system and to communicate the data. In the literature the use of wind turbines or airflow generators to power autonomous sensors are reported [33, 37]. The available theoretical airflow power can be calculated with the kinetic energy. Using the formula for the moving system the flow energy can be obtained:

(3) $$E=\frac{1}{2}mv^{2}=\frac{1}{2}\rho A\Delta tv^{3}$$

where ρ is the fluid density, A is the area normal to flux, v is the airflow speed and Δt is the observation time. The kinetic energy can be easy converted into the airflow power:

(4) $$P=\frac{1}{2}\rho Av^{3}$$

This power is function of air density, which can be assumed to be of 1.2 kg/m^3^ area, and airflow speed. The theoretical maximum quantity of energy for a standard area of 55 cm^2^ and a wind velocity 4.5 m/s is about 300 mW. A generator module cannot extract all of this power, since the relatively high viscous drag on the blades, the bearing losses and other factors. The wind power is corrected with a power coefficient less than unity (C_p_). Large-scale airflow generators can be highly efficient, with power coefficients greater than 0.5 being achievable; for small-scale airflow generators the performance is less good, about 0.1 [34].

**Figure 13.**
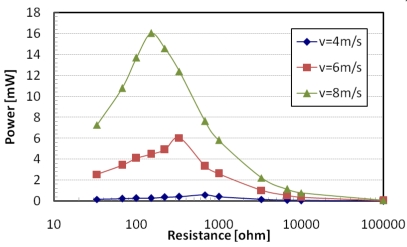
Power values of the tested airflow harvester for different airflow speeds and loads.

**Figure 14.**
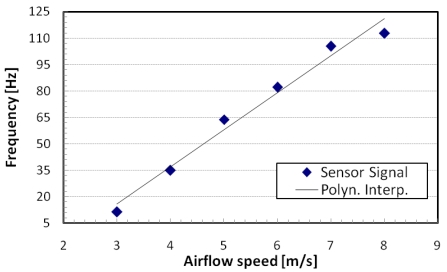
Characterization of the rotor frequency with different airflow speeds.

In the proposed application different airflow harvesters using commercial rotor and generator parts have been tested to study the power efficiency as a function of the load. The efficiency obtained was about 0.08. In Figure 13 a maximum power of 16 mW can be observed for a load of 150 Ω by a brushless generator and an impeller of 7 cm diameter. An experimental setup has been arranged to test the autonomous system by measuring the flux inside the pipe and the voltage that can be generated by the airflow harvester. The obtained results have shown a linear behavior of the sensor, as shown in Figure 14. The system begins working for airflow speed higher than 3 m/s, the measured data are stored into a non volatile memory and downloaded to a readout system. Depending on the power supply of the airflow generator, if the flow is not sufficient to supply the autonomous sensor, this can generate a black-out period. For this reason it is not able to record a trace of an absolute time of the measurement.

## 5. Conclusions

Autonomous sensors working without batteries and reducing the problem of environmental impact were presented and discussed. A classification of “passive autonomous sensors” and “self powered autonomous sensors” was introduced to distinguish between those that are just passive elements interrogated wirelessly from the front-end electronics and those that have a power harvesting system or are supplied by an electromagnetic field used as communication support and to obtain the electric power required to function properly. The general building blocks of these autonomous sensors were presented and the design guidelines that such a system must follow were given, along with different proposed applications of autonomous sensors applied in harsh or hermetic environments. Two examples of passive autonomous sensors were reported, the first one for humidity measurements which presents a distance interval of about 30 mm and the possibility to compensate the distance variation. The second one can measure high temperatures with a maximum limit of about 350 °C, guaranteeing the inviolability of the harsh environment. Furthermore, two applications of self-powered autonomous sensors with two different power harvesting modules are reported. The power harvesting modules allow the possibility of performing the measurement when needed, independently from the presence of the readout unit; thermal gradients were exploited by a thermoelectric converter as power harvesting source or airflow by an electromechanical generator. The reported examples have the same block diagram structure, but the modularity of such systems was applied for two different applications. The use of this kind of systems represents a good opportunity for remote environments for which small size, autonomous power supply, and ability to sense and transmit data are important characteristics.

## Figures and Tables

**Table 1.** Comparison of published power harvesters for autonomous sensors.

**Author and Reference**	**Generator Type**	**Energy**	**Generator Volume [cm^3^]**	**Power unprocessed [μW]**	**Power processed [μW]**	**Power Density [μW/cm^3^]**
**Michelson [34]**	Electrostatic	Motion	0.6	2.4	/	4
**Hammond [34]**	Piezoelectric	Motion	4.8	1700	700	145
**Ferrari [36]**	Piezoelectric	Motion	0.65	203	/	312
**Li [34]**	Electromagnetic	Motion	1	/	100	100
**Dalola [23]**	Thermoelectric	T Gradient	5.76	/	900	156
**Dalola [24]**	Thermoelectric	T Gradient	3.87	31800	/	8200
**Hande [33]**	Photovoltaic	Solar	40	400000	/	10000
**Weimer [37]**	Electromagnetic	Airflow	23	/	80-650	3.5-28
